# Posterior reversible encephalopathy syndrome in a patient treated with Ustekinumab^☆^

**DOI:** 10.1016/j.abd.2025.501177

**Published:** 2025-08-09

**Authors:** Marilda Aparecida Milanez Morgado de Abreu, Naíra M. de A. Guaresemin, Murilo de Oliveira Lima Carapeba

**Affiliations:** a Department of Dermatology, Hospital Regional de Presidente Prudente, Universidade do Oeste Paulista, Presidente Prudente, SP, Brazil; b Department of Radiology and Diagnostic Imaging, Hospital Rio Preto, São José do Rio Preto, SP, Brazil

Dear Editor,

Ustekinumab, a monoclonal antibody targeting the p40 subunit of Interleukin (IL)-12 and IL-23, is a safe and effective treatment for psoriasis.^1^ Although most patients do not experience serious adverse effects, some neurological conditions have been reported.^2^, ^3^, ^4^, ^5^, ^6^

This report describes a case of posterior reversible encephalopathy syndrome (PRES) caused by ustekinumab.

The study was approved by the Research Ethics Committee, number 78147224.3.0000.5515, and the patient signed the informed consent form.

A 47-year-old male patient with psoriasis vulgaris for 20 years presented with erythematous-scaly plaques covering 28% of his body surface area, with a Psoriasis Area and Severity Index (PASI) of 24.4. He had a history of alcoholism, grade II hepatic steatosis, and controlled arterial hypertension. He developed disease progression, previous intolerance to methotrexate, and no response to acitretin.

It was decided to initiate ustekinumab, 90 mg (dose adjusted to weight >100 kg), at weeks 0 and four, subcutaneously, and maintenance every 12 weeks. After the second dose (week six), the patient suddenly developed mental confusion. He was hospitalized, ustekinumab was suspended due to suspicion as the causative agent, and he was treated with risperidone, antihypertensives, promethazine, and lactulose. On the third day, he developed headache and tetraparesis.

A magnetic resonance imaging (MRI) of the brain revealed bilateral foci of hyperintensity in the periventricular and subcortical white matter of the frontal and parietal lobes on fluid-attenuated inversion recovery (FLAIR) and diffusion-weighted sequence (DWI) images, and foci of hypointensity in the reconstruction of the apparent diffusion coefficient (ADC) maps, characterizing restricted mobility of water molecules, and consistent with true restriction caused by ischemic foci (Fig. 1). The findings were consistent with the PRES diagnosis. Laboratory investigations ruled out infections and metabolic or inflammatory diseases, such as autoimmune ones, and it was impossible to rule out the occurrence of an epiphenomenon.

**Figure 1:**
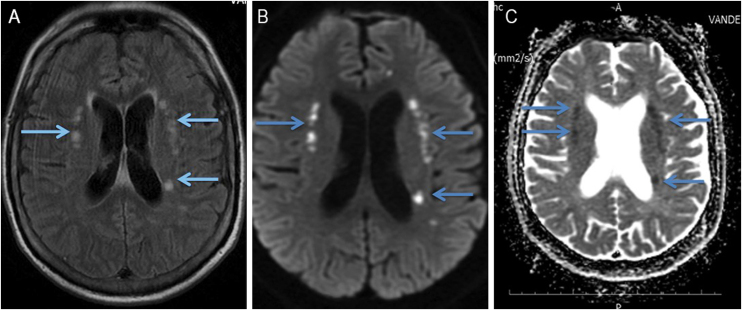
Magnetic resonance imaging of the brain. (a) FLAIR (fluid-attenuated inversion recovery) showing small foci of hyperintense signal in the periventricular and subcortical white matter of the bilateral frontal and parietal lobes. (b) DWI (diffusion-weighted imaging) showing hyperintense signal from the punctate foci seen on FLAIR (fluid-attenuated inversion recovery), suggesting cytotoxic edema. (c) ADC (apparent diffusion coefficient) MAP confirms the diffusion findings, showing restricted diffusion.

The neurological symptoms were reversed with supportive measures and he was discharged from the hospital on the 10^th^ day with oral memantine hydrochloride.

An MRI of the brain three months later showed the same hyperintense foci on FLAIR, with no changes in the DWI sequences and ADC map, corresponding to small areas of gliosis (scars).

One year later, the patient had a PASI of zero, with no neurological deficits.

Immunobiologicals represent an advance in the treatment of psoriasis and have been increasingly used.^1^ Serious side effects have been reported, mainly with anti-TNF-α. Ustekinumab, although safe, can also cause severe reactions, including neurological complications, such as PRES (Table 1),^1^, ^2^, ^3^, ^4^, ^5^, ^6^ this being the first case documented in Brazil and the seventh in the world. The development of PRES can occur soon after induction,^4^, ^6^ as in this case, or after years of using ustekinumab.^2^, ^3^, ^5^

**Table 1:** Characteristics of cases of Posterior Reversible Encephalopathy Syndrome (PRES) associated with ustekinumab reported in the literature.

Authors/Year	Age/Sex	Dose	Time of treatment from symptom onset	Indication	Clinical evolution: Persistent neurological deficit	Radiological evolution: Persistent radiological images
Gratton et al., 2011^2^	65/Female	45 mg every 12 weeks	2 1/2 years	Psoriasis	No	No
Dickson, Menter, 2017^3^	58/Male	90 mg every eight weeks	6 years	Psoriasis and psoriatic arthritis	No	No
Mishra, Seril, 2018^4^	18/Female	340 mg single induction dose	12 days following induction	Crohn’s Disease	No	No
Mishra, Seril, 2018^4^	54/Male	390 mg single induction dose	6 days following induction	Crohn’s Disease	No	No
Jordan, Kinnucan, 2022^5^	64/Female	90 mg every eight weeks	2 1/2 years	Crohn’s Disease	Difficulty walking	Yes (one month later)
Sarto et al., 2022^6^	48/Male	390 mg followed by 90 mg at week eight	30 days after the second dose	Crohn’s Disease	No	Yes (18 months later)
Este estudo	47/Male	90 mg at weeks 0 and four	14 days after the second dose	Psoriasis	No	Yes (three months later)

PRES is an uncommon, often reversible, severe neurological disorder with acute symptoms including headache, visual changes, paresis, nausea, altered consciousness, and seizures.^7^, ^8^

It seems to result from loss of cerebral vascular autoregulation or endothelial dysfunction, most commonly associated with malignant hypertension, eclampsia, and immunosuppressive agents such as corticosteroids, mycophenolate, cyclosporine, cyclophosphamide, and methotrexate.^6^, ^7^, ^8^ The name is imprecise, as it is not an alteration exclusive to the posterior brain regions and does not always lead to complete reversion.^6^, ^7^, ^8^

The MRI ensures the diagnosis and predicts prognosis, assessing whether or not the lesions are reversible. The classic findings are bilateral diffuse or focal symmetrical hyperintense signal on FLAIR images, predominantly in the white matter, involving the posterior regions, mainly the occipital and parietal lobes. Atypical images, including involvement of the frontal lobe, basal ganglia, temporo-occipital junction, and cerebellum, do not rule out the diagnosis.^7^

Most cases lead to neurotoxicity related to vasogenic edema, with no restriction on DWI sequence, and are reversible. However, approximately 11% to 26% of cases of infarction or tissue injury with cytotoxic edema, which is seen as restricted diffusion, evolving with sequelae ^7^ are described as in this patient, who evolved with permanent imaging lesions, perhaps due to a delayed diagnosis.^2^, ^3^, ^4^

There is no consensus on PRES treatment, but it is directed at the precipitating cause, in addition to management with antiepileptics. Symptoms usually resolve in one or two weeks.^9^

Despite the safety of ustekinumab and other drugs increasingly used in dermatological practice, the present case emphasizes their association with the occurrence of potentially serious neurological events. Early recognition and adequate management would prevent or reduce possible neurological sequelae and fatal outcomes.

## Financial support

None declared.

## Authors' contributions

Marilda Aparecida Milanez Morgado de Abreu: Approval of the final version of the manuscript; design and planning of the study; effective participation in research orientation; intellectual participation in the propaedeutic and/or therapeutic conduct of the studied cases; critical review of the literature; critical review of the manuscript.

Naíra M. de A. Guaresemin: Design and planning of the study; drafting and editing of the manuscript; collection, analysis, and interpretation of data; intellectual participation in the propaedeutic and/or therapeutic conduct of the studied cases; critical review of the literature.

Murilo de Oliveira Lima Carapeba: Design and planning of the study; drafting and editing of the manuscript; collection, analysis, and interpretation of data; intellectual participation in the propaedeutic and/or therapeutic conduct of the studied cases; critical review of the literature.

## Conflicts of interest

None declared.
